# Lung ultrasound: an additional tool in COVID-19

**DOI:** 10.1590/0100-3984.2020.0051

**Published:** 2020

**Authors:** Rodrigo Ribeiro de Oliveira, Thiago Potrich Rodrigues, Paulo Savoia Dias da Silva, Andrea Cavalanti Gomes, Maria Cristina Chammas

**Affiliations:** 1 Instituto de Radiologia do Hospital das Clínicas da Faculdade de Medicina da Universidade de São Paulo (InRad/HC-FMUSP), São Paulo, SP, Brazil.

**Keywords:** Lung ultrasound, Chest, Lung, COVID-19, Coronavirus, Pneumonia, Ultrasonography, Ultrassonografia pulmonar, Tórax, Pulmão, COVID-19, Coronavírus, Pneumonia, Ultrassonografia

## Abstract

Lung ultrasound is a well-defined diagnostic modality in the point of care emergency medicine concept. In the context of the coronavirus disease 2019 (COVID-19) pandemic, the lung ultrasound assumed an essential role in this disease, with a valid correlation of the imaging results with computed tomography. Recognize how the diagnostic possibilities of ultrasound in the approach of COVID-19 and its differential diagnoses are fundamental.

## Abstract (pt)

A ultrassonografia pulmonar é uma modalidade diagnóstica bem estabelecida no conceito *point of care* da medicina de emergência. No contexto da pandemia pela doença do coronavírus 2019 (COVID-19), a ultrassonografia assumiu um papel importante, apresentando boa correlação dos seus achados com a tomografia computadorizada. Conhecer as possibilidades diagnósticas da ultrassonografia é fundamental na abordagem da COVID-19 e seus diagnósticos diferenciais.

## INTRODUCTION

Lung and pleural ultrasound has become very important in the assessment of patients in emergency and intensive care units. Several protocols defined in the point of care ultrasound concept, such as dedicated ultrasound limited to a specific issue, are well described in the literature. For example, the use of the bedside lung ultrasound in emergency in acute respiratory syndrome (BLUE protocol) or the rapid ultrasound for shock and hypotension in circulatory shock (RUSH protocol). In addition, it is part of the renowned extended focus assessment sonography for trauma (e-FAST)^(1-4)^.

Ultrasound is free of ionizing radiation, dynamic, inexpensive, and can be performed at the bedside. In the context of highly transmissible infectious diseases, its performance involves the exposure of only one professional, unlike computed tomography (CT). These devices can be easily protected with plastic, reducing the risk of contamination, and facilitating sterilization processes^(5)^.

The 2019 novel coronavirus disease (COVID-19), which started in Wuhan, China, in December 2019^(6,7)^, has since spread to all continents (213 countries), with 3,090,445 cases and 217,769 deaths worldwide on 30 April 2020. More than 71,886 people in Brazil have been infected, with 5,017 deaths on this date^(8)^. The use of lung and pleural ultrasound has become greatly important in the propaedeutic of these patients^(5)^.

Several conditions can be evaluated by lung and pleural ultrasound, including consolidations, atelectasis, interstitial and alveolar edema, pneumothorax, and pleural effusion. These findings are not all characteristic of patients with COVID-19. Ultrasound is important both in the assessment of differential diagnoses and in the follow-up of patients with COVID-19^(1,3,6,9)^.

The ultrasound assessment of the lung is based on artifacts, such as A, B, and Z-lines. The common signs are also mentioned, with some using analogies to translate these artifacts in a mnemonic process. The objective here is to facilitate the understanding of these artifacts and to demystify pulmonary ultrasound. In this context, this study reviews the primary chest ultrasound findings, highlighting those commonly seen in patients with COVID-19, and also suggests standard ways of performing and reporting on the examination.

## HOW TO PERFORM THE ULTRASOUND EXAMINATION

**Figure 1:**
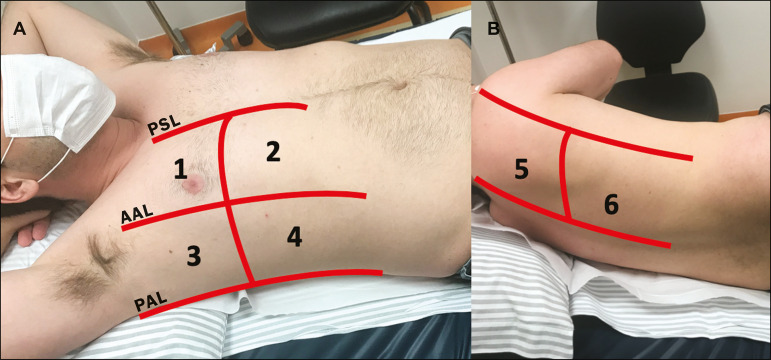
Patient positioning for the examination, anatomical references, and evaluated regions. **A:** Horizontal supine position. **B:** Left lateral position. PSL, parasternal line; AAL, anterior axillary line; PAL, posterior axillary line. 1, anterosuperior region; 2, anteroinferior region; 3, upper lateral region; 4, lower lateral region; 5, posterosuperior region; 6, posteroinferior region.

As ultrasound is a dynamic examination, the patient can be evaluated in the supine, lateral, prone, sitting, and standing positions. In the context of respiratory discomfort or airway control, the most common is to perform it in the supine or prone position, depending on the ventilatory strategy defined at the time (Figure 1).

The literature presents several protocols for different thoracic regions to be evaluated. The BLUE protocol evaluates six areas in each hemithorax, being the first protocol to involve the use of ultrasound for pleuropulmonary disorders. The anterosuperior and anteroinferior regions are evaluated on the anterior surface of the thorax (limited by the anterior parasternal and anterior axillary lines). The upper and lower lateral regions are evaluated on the lateral surface (limited by the anterior axillary and posterior axillary lines). Finally, the posterosuperior and posteroinferior regions are evaluated on the posterior surface (posterior to the posterior axillary line)^(1,4)^ (Figure 1).

Other protocols evaluate 14 areas in each patient (two anterior, two lateral and three posterior areas in each hemithorax), with the score depending on the findings^(9)^.

In general, the examination begins with the equipment adjusted for the abdominal ultrasound. Convex transducers can be used because their lower frequency and longer acoustic wavelength allow for the evaluation of deeper regions, but with a lower image resolution of the superficial plane. On the other hand, higher frequency linear transducers provide more details of the pleuropulmonary interface. Lichtenstein et al. state that the 6 MHz probe, used in their study is ideal^(1)^.

An image acquisition strategy in chest ultrasound (including the one used with COVID-19 patients) is to use a low mechanical index, starting at 0.7 and reducing it to improve image acquisition if necessary. It is recommended to avoid the phenomenon of saturation as much as possible, cosmetic filters, specific imaging modalities, and the use of a harmonic image since on chest ultrasound, the diagnosis is based on ultrasound artifacts.

The transducer should be preferably used in the longitudinal direction. This plane shows two acoustic shadows that represent artifacts posterior to the ribs. The transducer can also be used parallel to the ribs, showing a larger field. However, the rib references are lost. This acquisition is especially recommended when an abnormality is characterized. This plan, with a greater field of evaluation, can also be important in the diagnosis of pneumothorax^(10)^.

## NORMAL FINDINGS IN LUNGS AND PLEURAL ULTRASOUND

### Pleural line

The pleural line has a hyperechogenic ultrasound aspect and represents its parietal and visceral leaflets. As it is a dynamic examination, we note the pleural movement during respiratory incursions (lung sliding). It can be confirmed using an M-mode assessment, in which the characteristic finding is the “seashore sign”, which represents a good pleural sliding. The opposite is observed in the presence of pneumothorax, of a selective endotracheal tube, and, more rarely, of atelectasis^(11)^.

### A-lines

**Figure 2:**
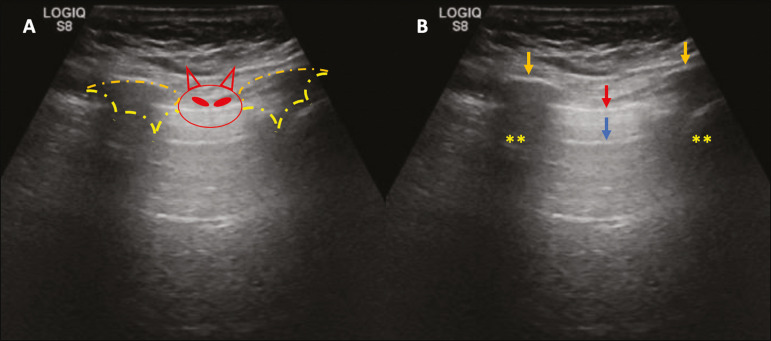
Chest ultrasound, longitudinal section in B-mode. **A:** “Bat sign”. **B:** Anatomical findings corresponding to the so-called “bat sign”. Ribs (orange arrows) and their respective acoustic shadows (asterisks). The pleura is hyperechogenic and identified between the ribs (red arrow). A-line (blue arrow) parallel to the pleura and originating from the pleural reverberation. The bat wings correspond to both the ribs and their acoustic shadows, and the bat head to the pleural echo.

**Figure 3:**
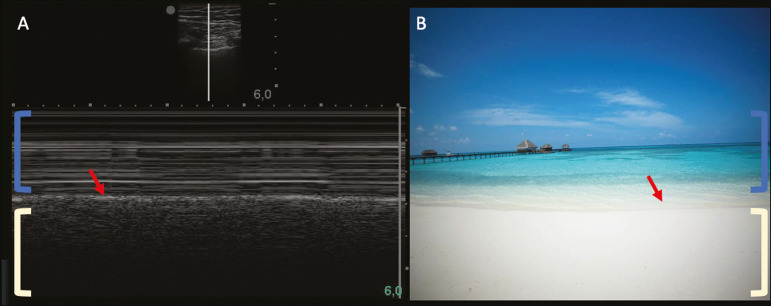
B-mode chest ultrasound shows the representation of two dimensions in the longitudinal section. **A:** Seashore sign. **B:** Analogy to “beach sand” and “sea wave” (seashore sign). Above the pleural line (arrows), the superficial tissues without movement are visualized in the form of lines parallel to the pleura, similar to “sea waves” (blue brackets). Below the pleural line, an artifactual image of the pleural sliding similar to “beach sand” (white brackets) is obtained. These findings are known as the “seashore sign”.

A-lines are formed due to a significant density difference between the pleura and the lung. The phenomena of pleural acoustic reverberation can be observed, with the formation of hyperechogenic lines. These lines are sparse and parallel to the pleural line, being repeated in the anteroposterior direction at the same distance from the pleura to the skin. This is a virtual finding, caused by an artifact resulting from pleuropulmonary interface characteristics^(12)^. In pathological processes, their characteristics can change, as shown below (Figures 2 and 3).

### B-lines

**Figure 4:**
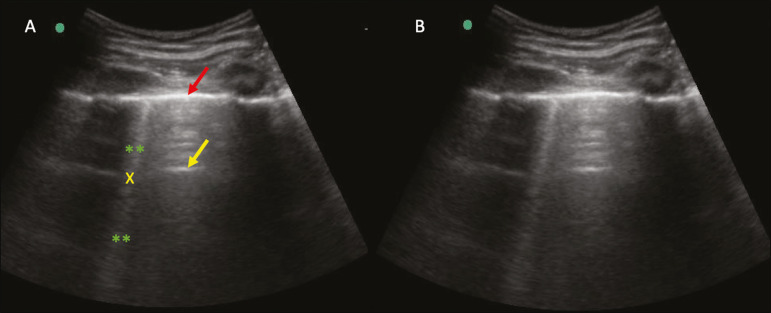
B-mode chest ultrasound. **A:** Representation with markings corresponding to the A, B, and pleural lines. The B-line (asterisks) represents vertical, hyperechogenic artifacts that cover the entire anteroposterior field analyzed. An important characteristic is that they eliminate the A-lines (yellow arrow) when crossing (X). The pleural line is shown as a hyperechogenic line (red arrow). **B:** Image demonstrates B-mode chest ultrasound without indicative markings.

**Figure 5:**
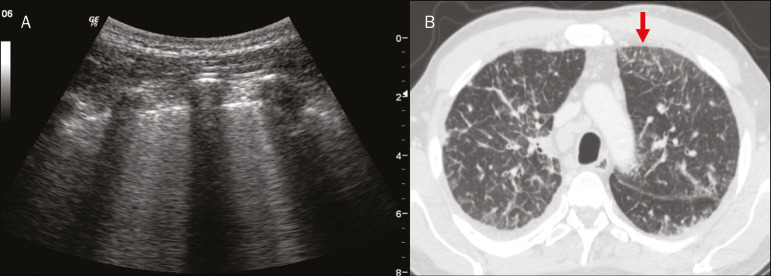
**A:** Ultrasound image shows coalescent B-lines (transducer in the left hemithorax, at the site of the arrow in **B**). **B:** Chest CT scan of the same patient shows thickening of the interlobular and intralobular septa due to carcinomatous lymphangitis, and some ground-glass opacities.

B-lines are hyperechogenic “comet-tail” artifacts of pleural origin that travel the entire anteroposterior extension of the evaluated field (perpendicular to A-lines) and eliminate A-lines^(13)^ during their trajectory. They represent thickened interlobular septa and may be present in cases of pulmonary edema, in addition to inflammatory processes (Figures 4 and 5). Patients bedridden for some time may have thickened septa, especially in the posterior regions (decubitus-dependent), which would lead to the presence of up to two B-lines per intercostal space, an aspect that still represents the absence of significant changes^(11,14)^.

### Z-lines

Z-lines (known as “false B-lines”) have no pathological significance. They are “comet-like” artifacts that start in the pleura and travel in the anteroposterior direction. However, they do not eliminate A-lines and do not reach the entire length of the evaluated pulmonary field. Unlike B-lines, Z-lines are not synchronous to respiratory incursions^(11)^.

## ULTRASONOGRAPHIC SIGNS OF PULMONARY, PLEURAL AND THORACIC WALL CHANGES

### Pathological B-lines

The presence of three or more B-lines per intercostal space represents the thickening of the subpleural interlobular or intralobular septa, which can be seen in conditions that affect the structures that cross such septa, such as the pulmonary interstitium, which contains veins, lymphatic vessels, and connective tissue^(12)^. Examples of such diseases are pulmonary edema, interstitial disorders, and lymphangitis.

**Figure 6:**
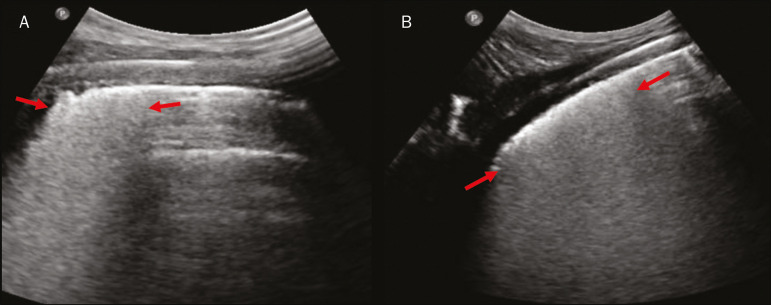
The chest ultrasound view demonstrates coalescent pleural B-lines in a patient with COVID-19. **A,B:** White lung appearance (arrows). On CT, this finding corresponds to ground-glass opacities.

Thicker B-lines (above 3.0 mm) are called coalescent B-lines, which correspond to ground-glass opacification in the pulmonary periphery, evident on CT^(13)^ . In the context of COVID-19, multiple coalescent B-lines on ultrasound configure a white lung pattern and correlate with ground-glass opacities identified on CT^(15)^ (Figure 6).

There is an inversely proportional correlation between the number of B-lines and the ratio of the partial pressure of oxygen/fraction of inspired oxygen^(16)^. That is, the greater the number of B-lines, the higher the gas exchange impairment. The use of ultrasound to control disease progression can be an auxiliary tool in monitoring such patients.

### Lung consolidation and C-lines

**Figure 7:**
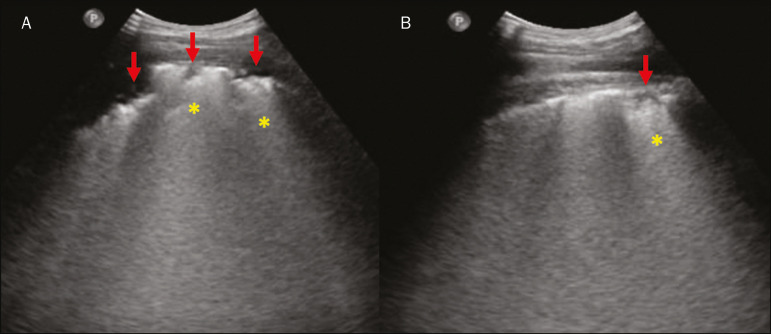
**A,B:** The chest ultrasound in a patient with COVID-19 demonstrates round small, hypoechogenic, and irregular subpleural consolidations (arrows). Pleural C-lines (asterisks) start from their posterior extremities, which are hyperechogenic and vertical, similarly to B-lines.

**Figure 8:**
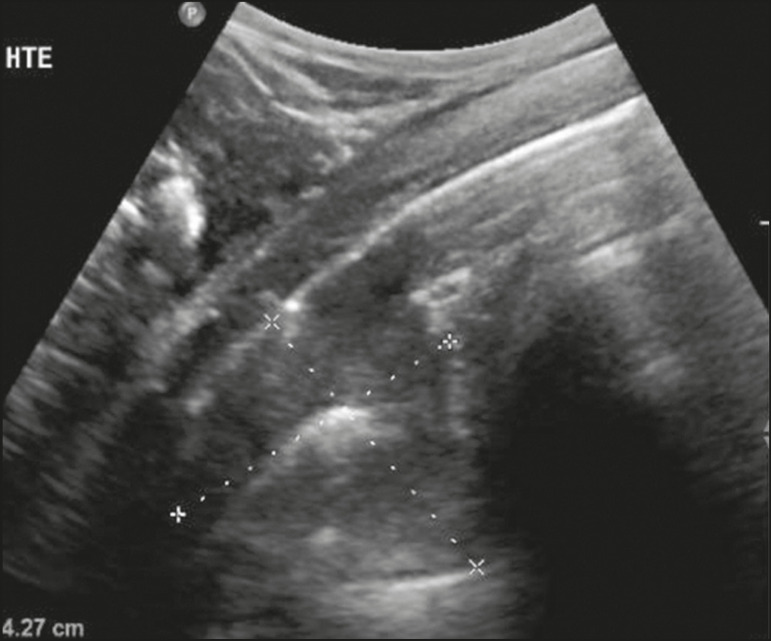
Ultrasonographic view of the left hemithorax shows subpleural consolidation. The echogenic aspect of a more extensive consolidation process resembles the liver texture, pulmonary hepatisation. The presence of dynamic air bronchograms corroborates the consolidation, differentiating it from pulmonary atelectasis.

Lung consolidation can present as hypoechogenic areas that touch the pleura, disfiguring the pleural echo in this region. The “comet-tail” artifacts at the base of the consolidations correspond to C-lines^(11,13)^ (Figures 7 and 8).

**Figure 9:**
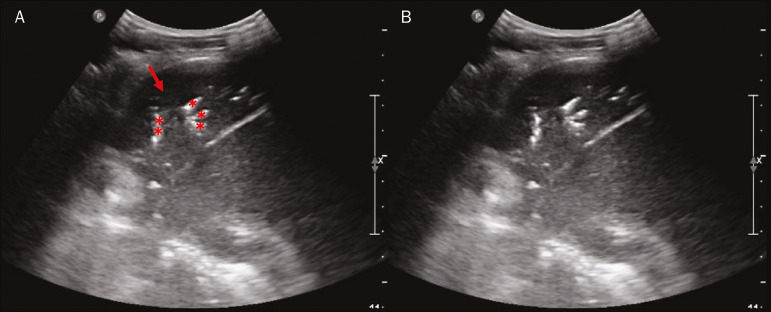
**A,B:** Schematic representation of dynamic air bronchograms. Dynamic air bronchograms (asterisks) on B-mode ultrasound (real-time assessment) with pulmonary consolidation (red arrow). Their characteristics change during the respiratory cycle phases. They may show signs of movement in a Doppler examination.

Air bronchograms are punctate or linear hyperechogenic images, whose characteristics change during the inspiration and expiration phases. They can be observed on ultrasound as real-time movements between the consolidations. The movement of air inside the bronchial tree within the consolidation is identified. In dynamic air bronchograms, a “vascular” pattern can be registered with a Doppler study^(17)^ (Figure 9).

**Figure 10:**
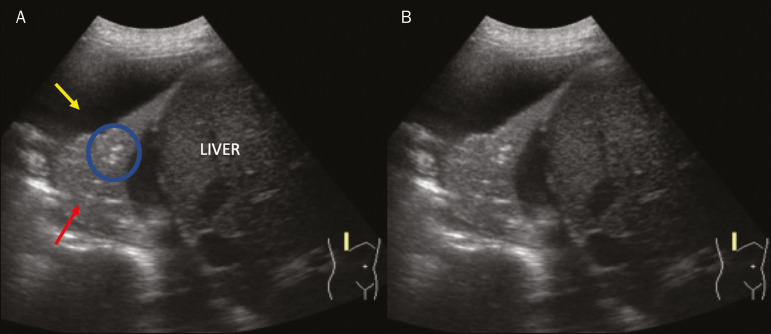
**A,B:** Ultrasound view of the lower third of the right hemithorax. Atelectasis (red arrow) and pleural effusion (yellow arrow). Note the volumetric reduction of the pulmonary segment assessed and the presence of static air bronchogram (circle). During respiratory incursions, there is no change in its characteristics and in the Doppler phenomenon, which would be present in the dynamic bronchograms of consolidations.

Pulmonary atelectasis is associated with pulmonary volumetric reduction, which may be similar to consolidations with air bronchograms. However, these are not usually dynamic but static. There is neither movement of hyperechogenic images between consolidations, nor presence of the “vascular” pattern on Doppler during the respiratory cycle. Dynamic air bronchograms can be visualized in up to 6% of atelectasis cases^(18)^ (Figure 10).

Pulmonary consolidations can give the lung parenchyma the aspect of “pulmonary hepatisation”, also called a tissular pattern, whose echogenicity is similar to that of the liver^(19)^ (Figure 8).

A meta-analysis study published in 2014 showed that in the clinical context, pulmonary ultrasound might have a sensitivity of 97% and specificity of 94% (CI 95%), with an area under the ROC curve of 0.99 in the diagnosis of pneumonia^(20)^.

In patients with COVID-19, consolidations can acquire a multi-lobular aspect and small dimensions, associated with dynamic air bronchograms^(21)^ (Figure 6). Massive pulmonary consolidations, as in lobar pneumonia, are not usually seen in COVID-19^(21)^.

### Pleural effusion

**Figure 11:**
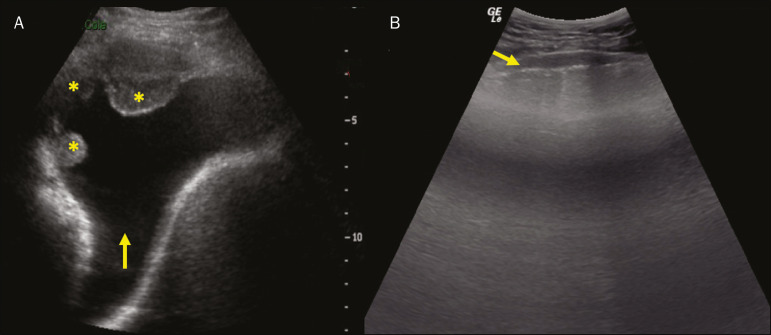
Other faces of the pleural effusion easily accessible on ultrasound. **A:** Multiple neoplastic nodules adhered to the pleura (asterisks) associated with pleural effusion (arrow). **B:** Pleural collection representing loculated pleural empyema (arrow).

The ultrasonic beam is easily transmitted through the liquid medium. Therefore, ultrasound can be used to assess pleural effusion, which generally has an anechoic appearance but may also be hypoechogenic. In M-mode, it is possible to verify the so-called “sinusoidal signal”, which occurs due to the floating movement of the lung in the liquid collection^(15)^. Small pleural collections (3 mL) can be detected by ultrasound^(22)^ (Figures 10 and 11).

The literature presents several ways to calculate and estimate the volume of the pleural collection. The distance between the visceral and parietal leaflets of the pleura can be measured with the transducer at the level of the posterior axillary line at the end of expiration. This distance can estimate the volume of pleural effusion. Thus, a distance of 3 mm would mean a volume of pleural effusion of 15 to 30 mL; 10 mm, volume of 75 to 150 mL; 20 mm, volume of 300 to 600 mL; and 35 mm, volume of 1,500 to 2,500 mL, respectively^(23)^. In addition, the estimated volume can be obtained by multiplying the distance between the pleural leaflets by 20, resulting in the estimated volume of the pleural collection^(24)^. It is worth mentioning that the volume of pleural effusion is not estimated using this method as there are significant differences between the estimates and actual volumes. This volume is only subjectively referred to as small, moderate, or large.

The anatomical site for pleural puncture can be chosen and marked with ultrasound. It must be marked with the patient in the position in which the puncture will be performed, while also observing the positioning of the upper limbs. The distance (thickness) between the skin and the pleural leaflet must be noted in the report, as well as the demarcated location.

In the context of COVID-19, the correct assessment of the pleural space is fundamental. Pleural effusion is a rare finding in patients with COVID-19 ^(23)^.

### Pneumothorax

Pneumothorax is the presence of air between the pleural leaflets, preventing adequate pleural sliding. Thus, the visceral pleura, which moves, will no longer be accessible. A-lines, on the other hand, might be present and originate from the parietal leaflet^(13)^.

**Figure 12:**
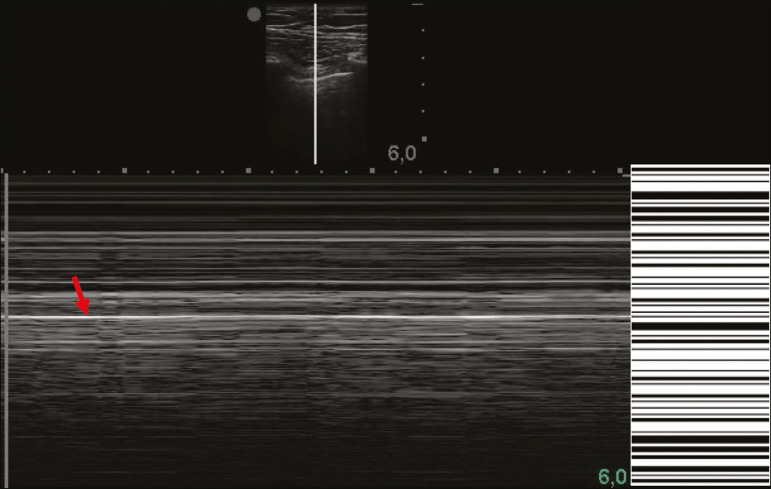
Pneumothorax. In M-mode, the “stratosphere” or “barcode sign” can be visualized (shown on the right side of the image), indicating the absence of pleural sliding. The “seashore sign” is no longer present. The hyperechogenic pleura is identified by the arrow. This finding may be present in other conditions such as selective intubation and diaphragmatic paralysis.

In M-mode ultrasound, the physiological aspect of the pleuropulmonary interface (represented by the “seashore sign”) is lost. In the presence of pneumothorax, the “stratosphere sign”, also called “barcode”, will be visualized, confirming the absence of pleural sliding^(1)^ (Figure 12).

The lung point can be found in B-mode, which is pathognomonic for pneumothorax. It is defined as the transition between the normal pleuropulmonary interface, in which physiological sliding is observed, and the region of the absence of sliding, representing the starting point of pneumothorax. In this case, the sensitivity of the examination is 66%, and the specificity is 100%^(25)^.

In COVID-19, as in the case of pleural effusion, pneumothorax is uncommon^(26,27)^. However, it is a possible complication during the treatment of patients on invasive mechanical ventilation.

### Subcutaneous emphysema

**Figure 13:**
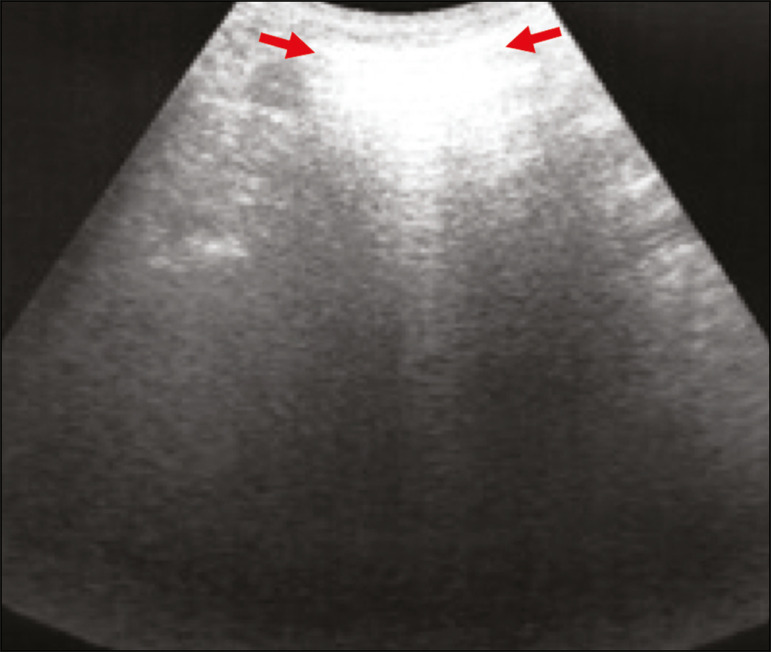
B-mode chest ultrasound demonstrates subcutaneous emphysema (arrows).

Subcutaneous emphysema is the presence of enclosed air in the subcutaneous tissue. With this condition, vertical E-lines are observed. These lines do not arise from the pleural line, but the subcutaneous tissue. As the air is in the subcutaneous tissue (that is, it does not move with breathing), these lines do not synchronize with the respiratory movements. E-lines are well defined and also eliminate A-lines, and can, therefore, be confused with true B-lines^(11)^ (Figure 13).

### Common findings in COVID-19

As reported by several authors, the basal superficial areas of the lungs are the most affected in COVID-19^(28)^. These regions can be easily assessed with ultrasound, making ultrasound an important auxiliary tool in COVID-19. However, there is no significant data in the literature about the sensitivity of ultrasound in the different degrees of pleuropulmonary involvement in COVID-19 to establish it as a screening method^(4,27)^.

Some findings on high-resolution chest CT show agreement with sonographic aspects. Thickening, pleural irregularity, and consolidations are well characterized in both methods.

**Figure 14:**
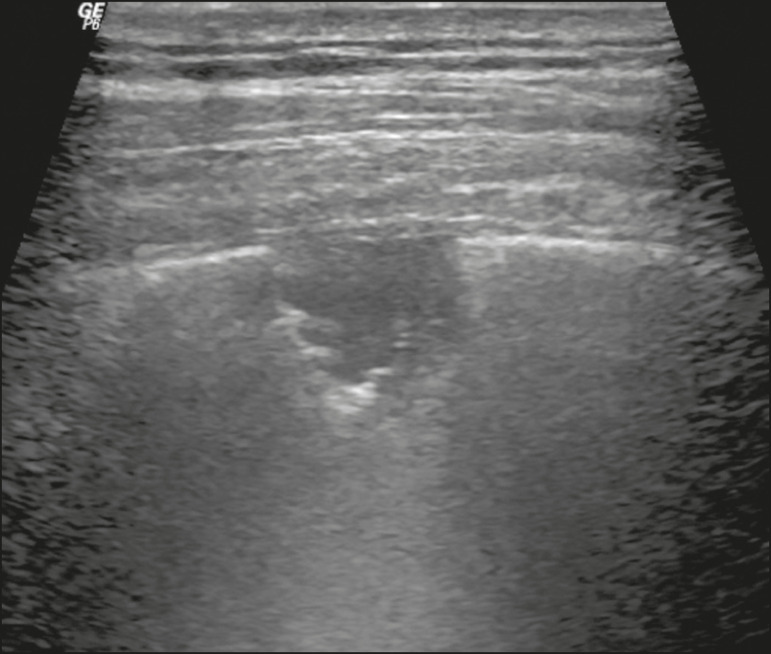
Chest ultrasound in a patient with COVID-19. The presence of pleural thickening, a hypoechogenic image with irregular contours, compatible with subpleural consolidation, less than 2.0 cm, and discrete C-lines are identified with the linear transducer in the intercostal space.

Ground-glass opacities on high-resolution CT correspond to multiple coalescent B-lines on ultrasound, sometimes with a white lung appearance, heterogeneously distributed across the lung parenchyma, in contrast to the homogeneous aspect of pulmonary edema^(6,22)^ (Figures 13 and 14).

**Figure 15:**
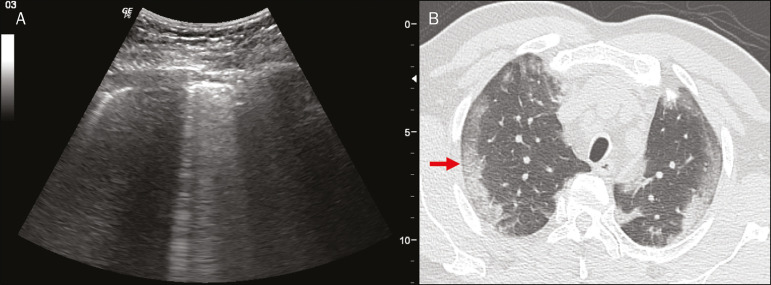
Chest ultrasound and CT in a patient with COVID-19: correlation between the findings. **A:** Coalescent B-lines characterized by the white lung appearance on ultrasound. **B:** On CT, peripheral ground-glass opacities (arrow) are identified at the same site.

**Figure 16:**
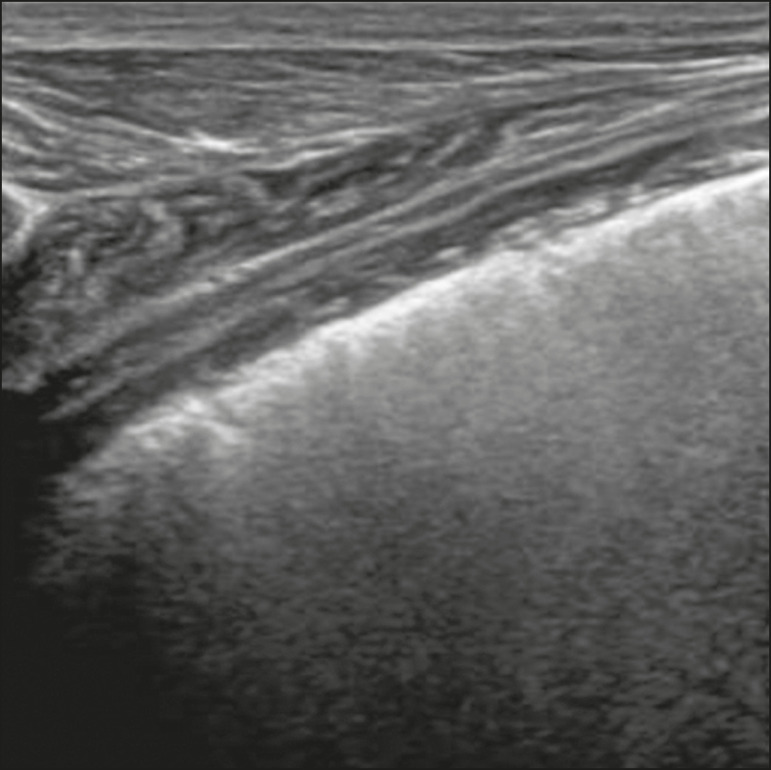
Chest ultrasound in a patient with COVID-19. Thickening and pleural irregularity, denote initial findings of the disease, can be observed using a highfrequency linear transducer.

The presence of scattered coalescent B-lines across the lung parenchyma, in addition to thickening and pleural irregularities, is common in the early stages^(6)^ (Figures 13, 15 and 16).

As the disease progresses (with a peak between nine and 13 days after the onset of symptoms), coalescent B-line areas increase on ultrasound, indicated by both increased ground-glass opacities and crazy-paving patterns on CT. In addition, there are consolidations, which are generally small and subpleural^(26)^.

## CHEST ULTRASOUND REPORT

Chest ultrasound reports should contain the main findings of each thoracic region evaluated, six in each hemithorax. It is important to identify them in each photographic record (Figure 1). In normal cases, it is recommended to report pleural integrity, regularity, and thickness, as well as the lung sliding and A-lines. It is important to verify the presence of pleural effusion, which is an uncharacteristic finding in COVID-19. The pathological findings and the predominant profile of each region evaluated should be reported so that any changes can be identified. If possible, each region should be compared with a previous ultrasound examination to characterize the evolution.

Findings such as pleural thickening, presence of more than two B or coalescent B-lines per intercostal space, and subpleural consolidations should be carefully evaluated in a patient with suspected or confirmed COVID-19, remembering that these findings are not pathognomonic for this condition.

### Report suggestion

#### CHEST ULTRASOUND

**1 - Technique:** Examination performed with a convex (low-frequency) and/or linear (high-frequency) transducer, with the patient in a horizontal dorsal and lateral decubitus and/or sitting position.
**2 - Lungs:** The intercostal spaces should be scanned, dividing each hemithorax into six regions, and the main pulmonary findings described using the table below:
  **Table:** 

Region	Right lung	Left lung
1. Anterosuperior		
2. Anteroinferior		
3. Upper lateral		
4. Lower lateral		
5. Posterosuperior		
6. Posteroinferior
1 - The following could be included in the above table:
A - Presence of only A-lines or up to two B-lines per intercostal space (normal aeration).
B1 - Presence of more than two B-lines per intercostal space (thickening of peripheral interlobular or intralobular septa).
B2 - Presence of coalescent B-lines (“ultrasound ground-glass opacities”).
C - Presence of small peripheral consolidations of less than 2.5 cm
CC - Presence of peripheral consolidations larger than 2.5 cm
CCC - Presence of lobar consolidation
AAA - Presence of pulmonary atelectasis
**Note:** If there are two or more changes in the same region, the most severe or most important for the clinical condition should be included. If it is important to report two changes in the same region, the model B2 + AAA, for example, can be used to indicate that there are coalescent B-lines and pulmonary atelectasis in the same region.
**3 - Pleura:** Any pleural thickening and/or irregularities should be described, and pleural spaces should be evaluated for virtual pleural spaces or presence of small/moderate/large right/left/bilateral pleural effusion. The effusion should be specified as a homogeneous liquid or containing septa/loculated/nodules. If pneumothorax is present, this should also be reported.
**4 - Diaphragmatic mobility:** Describe any reduction or paralysis of diaphragmatic mobility in one or both hemithorax.
**5 - Chest wall:** Report no significant changes or the presence of subcutaneous emphysema and/or costal arch fracture in a specific region.
**6 - Other findings:** Describe any findings in the examination, for example, pericardial effusion or significant changes in the thoracoabdominal transition, such as the caliber of the inferior vena cava, which is important in patients in hypovolemic shock^(3)^.
